# Ferroptosis: Redox Imbalance and Hematological Tumorigenesis

**DOI:** 10.3389/fonc.2022.834681

**Published:** 2022-01-26

**Authors:** Hongying Lan, Yu Gao, Zhengyang Zhao, Ziqing Mei, Feng Wang

**Affiliations:** ^1^ Key Laboratory of Molecular Medicine and Biotherapy, School of Life Science, Beijing Institute of Technology, Beijing, China; ^2^ School of Chemistry and Biological Engineering, University of Science and Technology Beijing, Beijing, China

**Keywords:** ferroptosis, redox, hematologic malignancies, lipid metabolism, iron homeostasis

## Abstract

Ferroptosis is a novel characterized form of cell death featured with iron-dependent lipid peroxidation, which is distinct from any known programmed cell death in the biological processes and morphological characteristics. Recent evidence points out that ferroptosis is correlated with numerous metabolic pathways, including iron homeostasis, lipid metabolism, and redox homeostasis, associating with the occurrence and treatment of hematological malignancies, such as multiple myeloma, leukemia, and lymphoma. Nowadays, utilizing ferroptosis as the target to prevent and treat hematological malignancies has become an active and challenging topic of research, and the regulatory network and physiological function of ferroptosis also need to be further elucidated. This review will summarize the recent progress in the molecular regulation of ferroptosis and the physiological roles and therapeutic potential of ferroptosis as the target in hematological malignancies.

## Introduction

**Figure 1:**
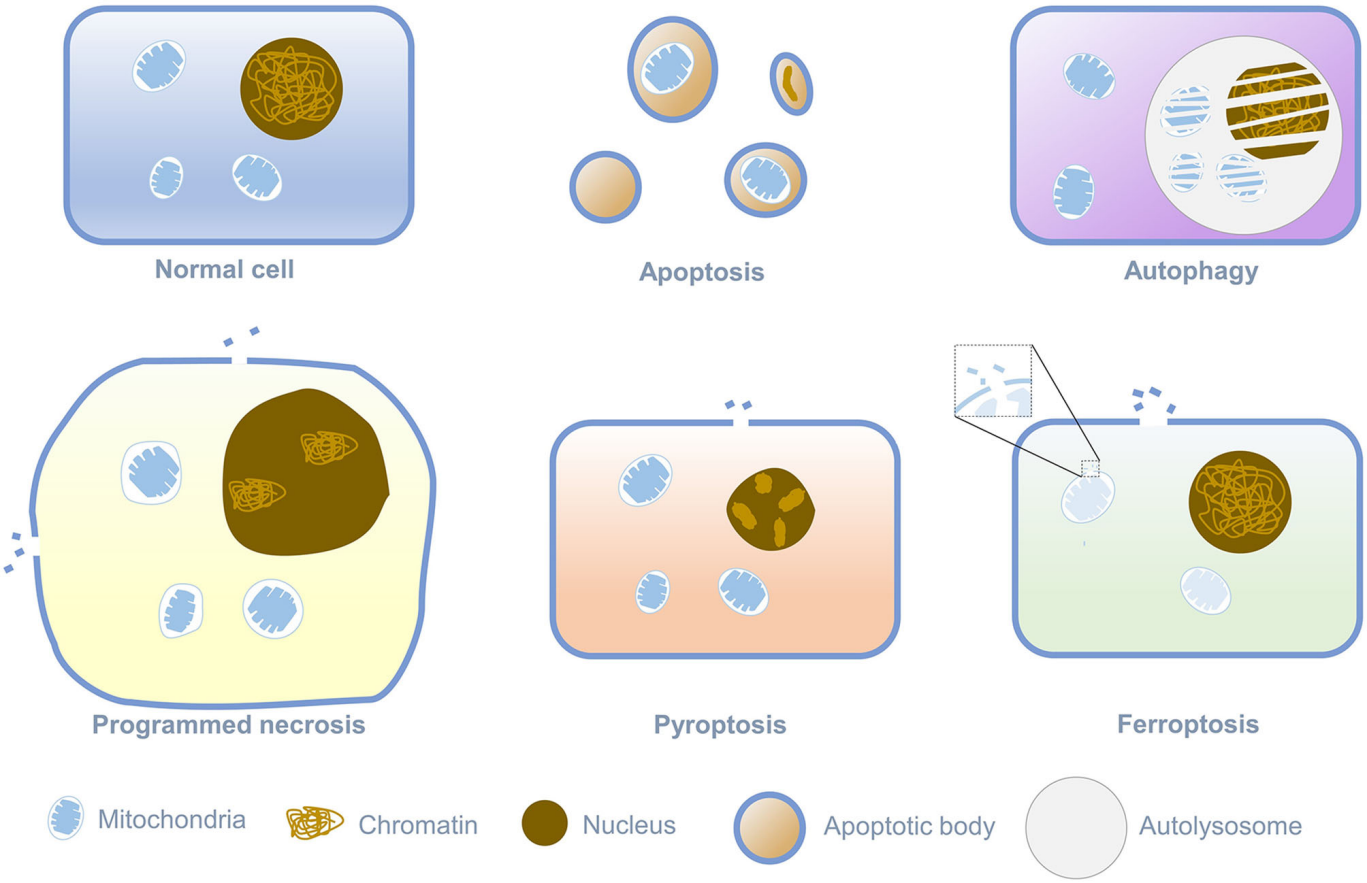
Morphological Features of different programmed cell death.

Death is an irreversible regulation process in the living cells, and different ways of death relate to the distinct physiological functions. Cell death is divided into programmed cell death and cell necrosis. Programmed cell death is an actively induced and tightly controlled process of cell suicide in response to various signal stimuli. In contrast, cell necrosis is an acute, spontaneous and passive death caused by unrepairable stress under pathological conditions, such as physical, chemical, hypoxia, or insufficient energy (mainly ATP) (1–6). When the cell is necrotic, the integrity of the cytoplasmic membrane is destroyed, and the barrier function to Na^+^, Ca^2+^, and water will be lost. Water flowing into the cell could lead to cytoplasmic swelling and nucleus pyknosis, eventually leading to cell rupture (7). The most canonical way of programmed cell death is apoptosis, which relates to cell contraction, chromatin condensation, and the formation of apoptotic bodies. With the continuous advancement of research on the manner of cell death, it has been found that in addition to apoptosis, programmed cell death also comprises autophagy, programmed necrosis, pyroptosis, and ferroptosis. Ferroptosis is a new form of cell death discovered by Stockwell in searching for small molecules targeted at RAS protein mutations related to cancer. Its morphological characteristics are different from any known form of cell death, as shown in **Figure 1** (8). Iron-dependent cell membrane lipid peroxidation will lead to ferroptosis. At this time, the cell’s mitochondrial membrane density will increase, while the mitochondrial cristae will decrease or disappear, and the mitochondrial outer membrane will rupture, but the nucleus will remain normal (9, 10). Since ferroptosis was defined as a new form of cell death in 2012, more and more researchers have garnered significant attention on ferroptosis and continuously identified the correlation of ferroptosis with cancer and tumor immunity. This review will summarize recent progress on the regulatory mechanism of ferroptosis and the pathological manifestations related to ferroptosis and propose potential treatment strategies.

## Mechanism of Ferroptosis

### Lipid Peroxidation Leads to Ferroptosis

The most apparent feature of ferroptosis in cells is lipid peroxidation. In the process of ferroptosis, the initial and essential step is that ACSL4, as a member of the acyl-CoA synthase long-chain family (ACSL) family, specifically catalyzes polyunsaturated fatty acid (PUFA), such as arachidonoyl (AA) and adrenoyl moieties (AdA), to form a long-chain acyl-CoA, namely PUFA-CoA (11). Downregulation of ACSL4 expression or pharmacological inhibition of ACSL4 activity (thiazolidinediones or triacsin C, etc.) can prevent ferroptosis (12–14). Lysophosphatidylcholine acyltransferase 3 (LPCTA3) then selectively uses phosphatidylethanolamine (PE) or phosphatidylcholine (PC) located on the endoplasmic reticulum as the receptor for acylated PUFA to generate PUFA-PE or PUFA-PC (15). Lipids with unsaturated fatty acids are typical peroxidation targets because carbon-carbon double bonds are susceptible to reactive oxygen species (ROS). According to different peroxidation mechanisms, lipid peroxidation can be divided into enzymatic and non-enzymatic types. It is currently considered that the enzyme pathway is mainly accomplished by lipoxygenase (LOX), which is a class of dioxygenases containing non-heme iron and can directly catalyze the peroxidation of PUFA-PE (13, 16, 17). Six LOX species have been identified in the human genome and referred to as 5-LOX, 12-LOX, 15-LOX-1, 15-LOX-2, 12R-LOX, and eLOX3 according to their oxidation positions on the arachidonic acid carbon chain (11, 18). When LOX is overexpressed, cells appear to be sensitive to ferroptosis, whereas inhibiting LOX activity, in turn, protects cells from RSL3-induced ferroptosis (19). The enzymatic lipid peroxidation is the reaction where enzymes specially select and catalyze substrates to generate the products. In non-enzymatic lipid peroxidation, free and unstable ferrous ions react with hydrogen peroxide to generate ferric ions and strongly oxidizing hydroxyl radicals (OH.). Hydroxyl radicals abstract the first hydrogen in the PUFA and form resonantly stable carbon-centered lipid radical (PUFA-R.), which can react with molecular oxygen to form lipid hydroperoxyl radical (PUFA-ROO.). Another hydrogen can be extracted by lipid peroxidation radical from the adjacent unsaturated fat chain, leading to the formation of lipid peroxides (PUFA-ROOH) and new resonant carbon center radicals. In this cycle, the chain reaction continues to proceed and generates new lipid peroxides until the concentration of PUFA-ROO is high enough for two PUFA-ROO to contact each other so that a new bond is formed (20–22). Lipid peroxidation can generate unstable hydroperoxyl groups in PUFA and promote oxidative truncation of PUFA-ROOH, creating electrophiles such as aldehydes and Michael receptors. The electrophilic products then attack proteins on the cell membrane, causing plasma membrane rupture and cell death (23, 24).

As a natural fat-soluble antioxidant, α-tocopherol can disrupt the chain reaction in lipid peroxidation and inhibit ferroptosis, owing to its high affinity for unpaired electrons (25, 26). Liproxstatin-1 and ferrostatin-1, two ferroptosis inhibitors identified by high throughput screening, have the characteristics of free radical-trapping antioxidants (RTA), and hence preventing ferroptosis by scavenging free radicals (8, 27, 28). Recent studies have demonstrated that cytochrome P450 oxidoreductase (POR) and cytochrome B5 reductase 1 (CYB5R1) can transfer electrons from NAD(P)H to downstream proteins such as cytochrome P450 (CYP), which incorrectly transfer electrons to molecular oxygen to generate hydrogen peroxide. The Fenton reaction between hydrogen peroxide and ferrous ions can induce ferroptosis (29). When intracellular expression of POR or CYB5R1 was downregulated, the H_2_O_2_ content was reduced, while the cell survival rate was remarkably increased (29, 30).

### Lipid Peroxidation Defense System

**Figure 2:**
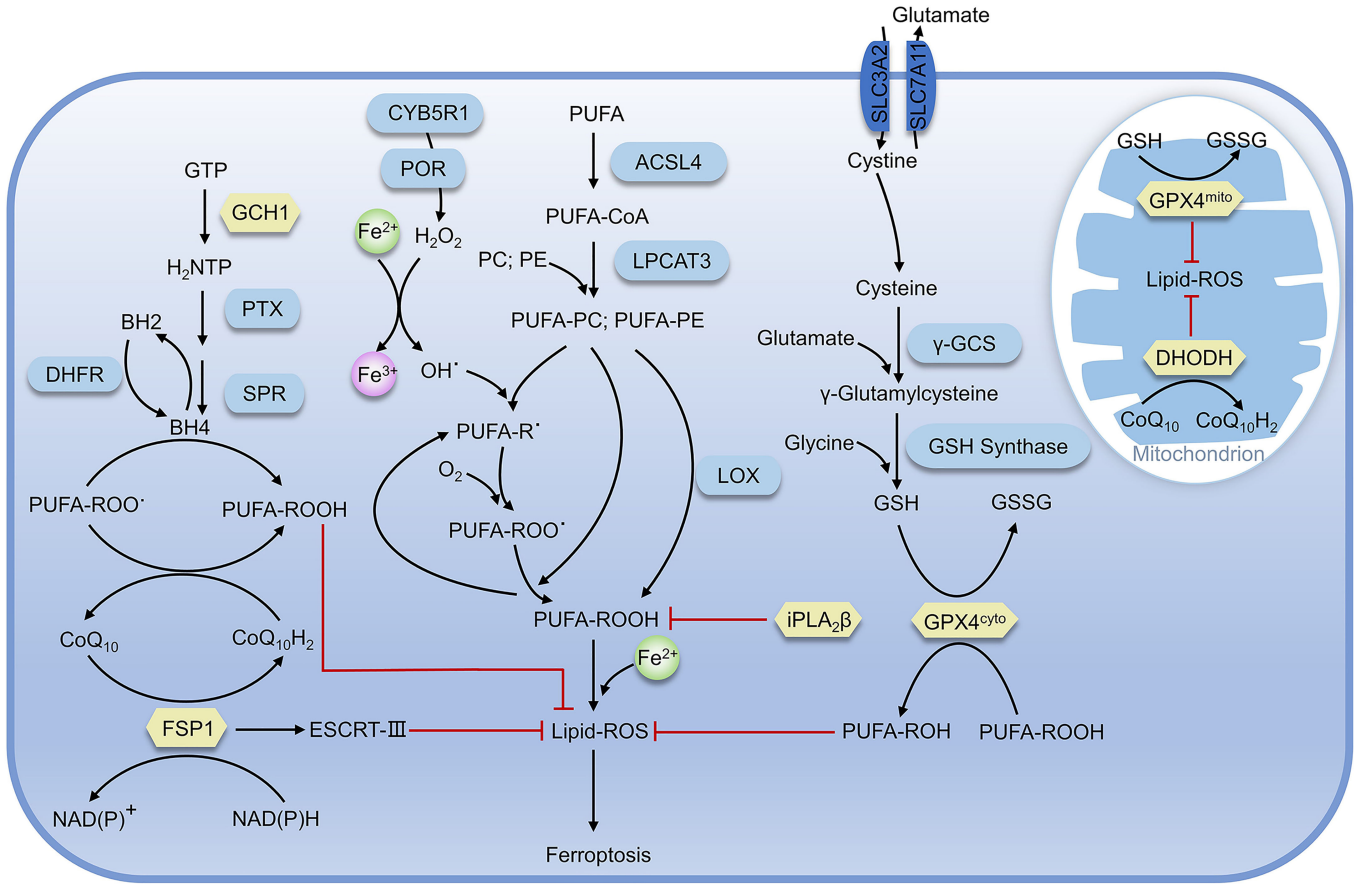
Regulatory pathways of ferroptosis. The proteins in yellow are key defense systems; the red line represents inhibiting effect (as in **Figure 4**).

As lipid supports the structure of the cell membrane or organelle membrane, lipid peroxidation can significantly change the physical properties of the lipid bilayer. As a marker and necessary prerequisite of ferroptosis, the accumulation of lipid peroxidation is regulated by various redox systems in cells (**Figure 2**) (21).

#### GPX4-GSH System

As a negative regulator for ferroptosis, GPX4 utilizes two reduced glutathione molecules (γ-glutamyl-cysteinyl-glycine, GSH) as the electron donor to reduce lipid peroxides (such as AA-OOH) into corresponding alcohols (AA-OH) and produce a molecule of oxidized glutathione (GSSG), reducing lipid peroxidation and preventing ferroptosis (31, 32).

The production of GSH in cells is inseparable from the system Xc^-^, a heterodimer composed of SLC3A2 and SLC7A11, which can execute the antiport of cystine and glutamate on the cell membrane, namely takes one molecule of cystine into the cell and releases one molecule of glutamate from the cell (33). Cystine transported into the cell is rapidly reduced to cysteine, which is involved in GSH synthesis and other physiological reactions. The γ-glutamylcysteine synthase (γ-GCS) performs the first and rate-limiting step in the process of GSH synthesis: synthesis of L-γ-glutamylcysteine from L-cysteine and L-glutamate in the presence of ATP, while GSH synthase catalyzes the synthesis of GSH from glycine and γ-glutamylcysteine (34, 35). After erastin treatment, ferroptosis-inducing agents56 (FIN56) can inhibit GPX4 and cells exhibit susceptibility to ferroptosis. When GPX4 is overexpressed, FIN56-induced ferroptosis would be impeded (36). Similarly, dihydroartemisinin (DHA) can also promote ferroptosis in glioblastoma through targeted downregulation of GPX4 and accumulation of lipid peroxidation (37). Albeit GPX4-deficient hematopoietic stem cells are prone to suffer from ferroptosis *in vitro*, GPX4-deficient mice still retain general function, owing to the antioxidant function of lipophilic vitamin E. Moreover, GPX4 combined with vitamin E can also prevent hepatocellular disease (38, 39).

#### FSP1-CoQ_10_ System

FSP1-CoQ_10_ system can antagonize ferroptosis in GPX4-GSH system-independent mechanism. Doll and Bersuker’s teams almost simultaneously demonstrated that FSP1 was strongly associated with ferroptosis (40, 41). FSP1 mainly contains two distinct structural domains, the N-terminal short hydrophobic region and FAD-dependent NAD(P)H redox region, and participates in ferroptosis through reduction of ubiquinone (also known as coenzyme Q_10_, CoQ_10_) and formation of panthenol (CoQ_10_H_2_), which is a reduced form of the fat-soluble antioxidant ubiquinone and can trap and reduce lipid peroxidation radicals (42). In addition, the overexpression of FSP1 in most tumor cells, and the treatment with the inhibitor of FSP1 (iFSP1), can make cells sensitive to RSL3 (43). Meanwhile, Bersuker et al. identified that FSP1 was a negative regulator of ferroptosis in screening for the synthesis of lethal CRISPR-Cas9, and the localization of FSP1 to the plasma membrane by myristoylation was necessary for its inhibition of ferroptosis (31). Subsequent studies demonstrated that FSP1 plays a role in membrane repair and resistance to ferroptosis in the panthenol-independent and ESCRT-III-dependent manner (44). Knockdown of FSP1 inhibits the expression of ESCRT-III subunits (CHMP5 and CHMP6), but the exact mechanism remains unclear. Furthermore, mice with knockout of the FSP1 gene did not show any abnormalities before at least one-year-old (45). In conclusion, promoting ferroptosis in tumor cells *via* targeting inhibition of FSP1would be a potential strategy for cancer therapy. Consistently, the development of FSP1 inhibitors is also of great significance for clinical research.

#### GCH1-BH4 System

Human GCH1 is a 270 kD complex composed of five dimers that catalyzes the conversion of guanosine triphosphate (GTP) to dihydroneopterin triphosphate (H2NTP), which is the first and rate-limiting step in the biosynthesis of BH4 (46). H2NTP was then transformed into BH4 by 6-pyruvoyl tetrahydrobiopterin synthase (PTS) and sepiapterin reductase (SPR). Kraft found that GCH1 was related to ferroptosis through CRISPR-Cas9 overexpression screening. GCH1 prevents the peroxidation of phosphatidylcholine with two polyunsaturated fatty acid chains to prevent ferroptosis through the antioxidant action of BH4, and BH4 may also be involved in the pathway of FSP1-CoQ_10_ as a biosynthetic precursor of CoQ_10_ (47). Dihydrofolate reductase (DHFR) participates in the regulation of ferroptosis by regenerating oxidized BH4 and the combination of inhibition of DHFR by methotrexate (MTX) with inhibition of GPX4 by RSL3 can promote ferroptosis (48, 49). In summary, BH4 is the core element of this system for ferroptosis resistance. In addition to GCH1 and DHFR, there are other factors mediating ferroptosis through BH4, and the imbalance of BH4 levels may be associated with the occurrence of ferroptosis-related diseases.

#### iPLA_2_β

Ca^2+^-independent phospholipase A_2_β (iPLA_2_β) can specifically hydrolyze sn-2 acyl bonds of phospholipids, which has been recently identified as a regulator of ferroptosis. When ferroptosis occurs, the characteristic product of lipid peroxidation, 15-hydroperoxy-arachidonoyl-phosphatidylethanolamine (15-HpETE-PE), is hydrolyzed by iPLA_2_β, which impedes subsequent ferroptosis-related effects. Decreased iPLA_2_β expression in cells was significantly more sensitive to RSL3-induced ferroptosis and showed higher ferroptosis markers associated with PE (50). The interaction of iPLA_2_β with various membrane substrates was simulated by computational modeling, and it was found that 15-HpETE-PE was more exposed to the membrane surface, close to the catalytic site (50). When the R747W mutation occurred in the catalytic domain, the interaction with the membrane was diminished, leading to a decline in the catalytic capacity of 15-HpETE-PE and the inhibition of ferroptosis in cells. It is demonstrated that the reduced enzyme activity may be linked to neurological diseases, including Parkinson’s disease in particular. Besides, iPLA2β was further identified as a regulator of p53-mediated ferroptosis, independent of GPX4 and FSP1, inhibiting ferroptosis by eliminating AlOX12-catalyzed lipid peroxidation. Notably, like FSP1, the lack of iPLA2β has no impact on cell function or tissue development, suggesting that it may be a potential target for inducing ferroptosis in tumor cells (51).

#### DHODH

Mitochondria is the indispensable organelle in eukaryotes where oxidative phosphorylation, energy generation, and important other functions such as signal transmission and energy metabolism occur. Mao et al. recently demonstrated that dihydroorotate dehydrogenase (DHODH) mediated the regeneration of panthenol in mitochondria to restore peroxide-damaged mitochondrial lipids and inhibition of ferroptosis by DHODH was independent of DHODH’s function in pyrimidine synthesis (52). DHODH can also produce panthenol to repair peroxide lipids, but DHODH targets mitochondrial membrane lipids instead of cytoplasmic lipids. GPX4 is subdivided into cytoplasmic and mitochondrial types, which are referred to as GPX4^cyto^ and GPX4^mito^, respectively. GPX4^mito^ and DHODH protect the mitochondria against oxidative damage independent of GPX4^cyto^ and FSP1. Mao also demonstrated that Brequinar, an inhibitor of DHODH, impeded the proliferation of tumor cells with low GPX4 expression. In addition, combined use of ferroptosis inducers could inhibit the growth of tumor cells with high GPX4 expression, implying a new approach for cancer treatment (52).

## Metabolic Regulation of Fe^2+^

Iron is a vital transition metal with redox activity in the body, which has implications in biological processes such as oxidative phosphorylation, DNA synthesis, and cell signaling (53, 54). Excessive or inadequate iron levels can lead to the loss of protein function, abnormalities in intracellular signaling, as well as out-of-control of metabolic networks, thus interfering with normal physiological processes (55). Ferrous ions are reported to be involved in the Fenton reaction and promote lipid peroxidation when ferroptosis occurs. In addition, reactive oxygen species (ROS) that trigger the Fenton reaction are also related to iron (56).

In a natural evolution, organisms have evolved multiple regulatory pathways of iron homeostasis. Most of the ferric ions in nutrients are absorbed by the brush border of duodenal cells, reduced to ferrous ions by duodenal cytochrome b (Dcytb), then transported to intestinal cells by Divalent metal ion Transporter 1 (DMT1) (57). In intestinal cells, a part of ferrous ion is fixed by ferritin, while the other parts are utilized by the ferroportin (FPN, also known as SLC40A1) and transported into the blood. Ferrous ions are oxidized to ferric ions in the blood by Hephaestin (HEPH) or Ceruloplasmin (CP) (53, 58). Transferrin (Tf) can bind two ferric ions and enter into cells through endocytosis after forming a complex with transferrin receptor (TfR). In the acidic environment of the Endosome, ferric ions are dissociated from Tf and reduced to ferrous ions by a six-transmembrane epithelial antigen of the prostate 3 (STEAP3), and then are transported to the cytoplasm by DMT1, while Tf and TfR can be recycled for the next transfer (57, 59, 60). Ferric ions entering the cytoplasm can function in the various physiological processes or stay in ferritin.

**Figure 3:**
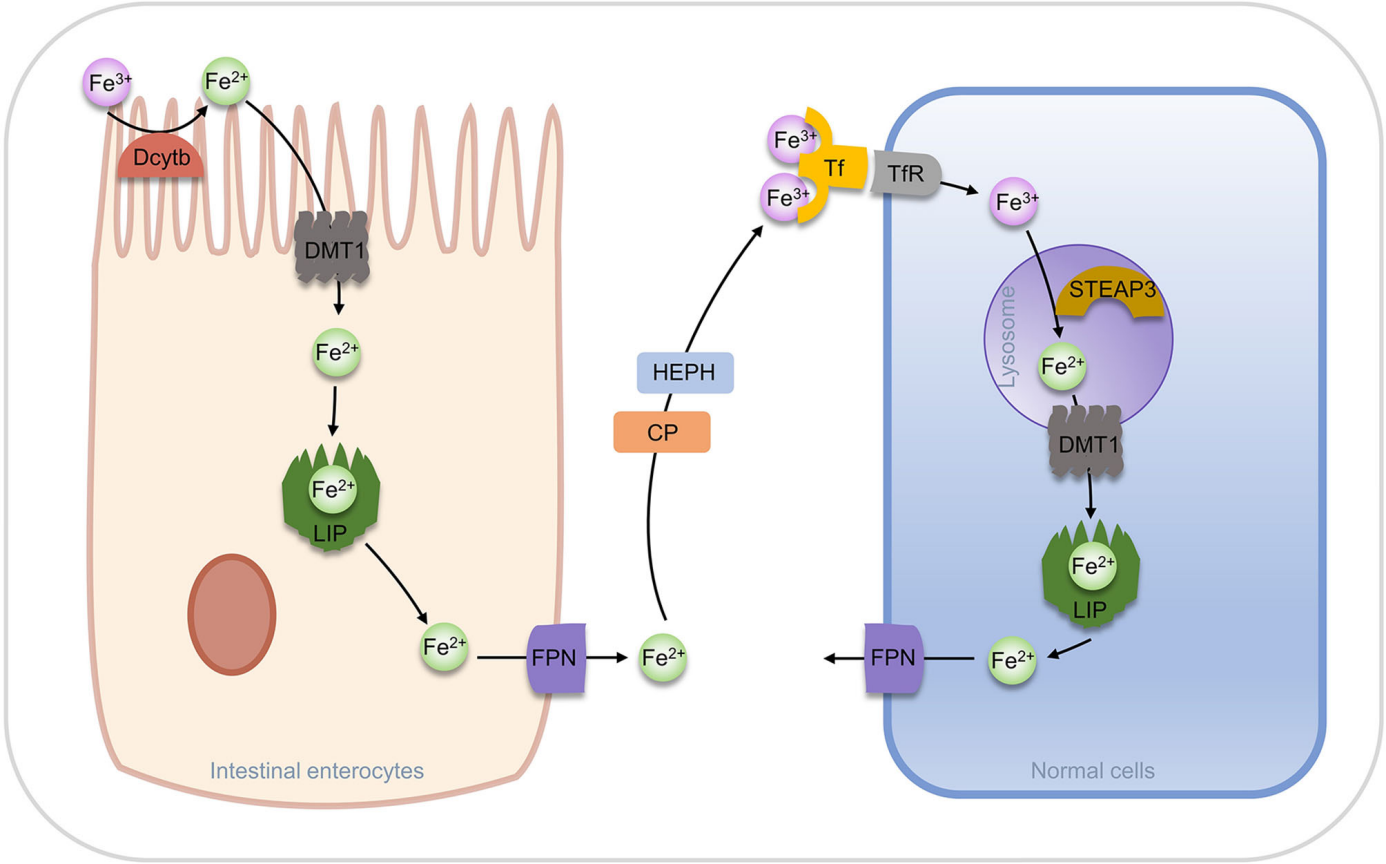
Regulation of iron transport *in vivo*.

Increased TfR expression was found in erastin-induced ferroptosis and p53 induced ferroptosis, suggesting that TfR is positively associated with ferroptosis (61, 62). In addition, it has been found that knockdown of FPN in neuroblastoma promotes ROS-dependent ferroptosis, and the downregulation of FPN expression leads to ferroptosis in the hippocampal area of rats and other diabetic cognitive dysfunction (63, 64). The storage of iron ions in cells is undertaken by ferritin, which is composed of a heavy chain (FTH) and a light chain (FTL) and forms a “labile iron pool (LIP)” of 12 or 24 polymers through the weak interaction. The storage pool can store more than 4500 ferrous ions. Ferritins from different species are distinct in ferritin size, amino acid sequence, iron access channel, and iron-binding site. However, from the aspects of shape and structure, they are all “pools” formed by orderly arranged helixes (65, 66). The iron storage pools control the concentration of free ferrous ions in the cytoplasm, which determines whether it acts as a beneficial cofactor or as a toxic-free radical catalyst in the cell and is key for the fate of the cell (**Figure 3**).

Nuclear Receptor Coactivator-4 (NCOA4) mediates the degradation of ferritin in an autophagy-dependent pathway where iron is isolated from ferritin and the accumulated free ferrous ions in the cytoplasm promote erastin-induced ferroptosis (67). Nuclear factor-erythroid 2-related factor 2 (NRF2) regulates the expression of FPN1, FTH, and FTL at the transcriptional level and promotes the stability of the intracellular iron environment (68). TfR and ferritin translation regulation at the transcriptional level in a cell operates through IRE-IRP. Iron regulatory proteins (IRPs) can be classified into IRP1 and IRP2. IRP1 can be converted between apo-form and holo-form, wherein the former can bind mRNA, and the latter has aconitase activity and is the main active form of IRP1 (69, 70). IRP2 is widespread in mammals and is the main executor of IRE-IRP iron regulation (69, 71). When the intracellular iron concentration is inadequate, IRP will bind to the 3’ untranslated regions of mRNA (UTR) of TfR with a high affinity and specific manner to enhance the stability of the mRNA and the expression level of TfR, resulting in increased iron transport into the cell (72). When intracellular iron is abundant, IRP binds to the 5’ UTR of ferritin and TfR, preventing their translation. IRP is at the control of intracellular iron level and H_2_O_2_, oxygen concentration, and oxidative stress signal (72, 73).

## Regulatory Mechanisms Associated With Ferroptosis

### Effect of Ether Phospholipid on Ferroptosis

Unlike typical fatty acids, which are connected to the glycerol framework *via* two ester bonds, the sn-1 position of ether phospholipids (ePLs) is linked to the aliphatic chain *via* the ether bond, while the position of sn-2 is linked to the polyunsaturated fat chain by an ester bond (74, 75). Carbons adjacent to ether bonds can be bonded in two ways: the first is a carbon-carbon single bond to form an alkyl ether; another is a carbon-carbon double bond for the formation of vinyl ethers (also known as acetal phospholipids). Zou et al. found that peroxisome composition was associated with ferroptosis using genome-wide CRISPR-Cas9 suppressor screens, in combination with the protein network database STRING and the pathway analysis algorithm they developed. Furthermore, they found utilizing lipidomics that polyunsaturated ether phospholipids (ePLs) generated by the peroxisome pathway act as substrates of lipid peroxidation to induce ferroptosis (76). When the expression of ether phospholipid-related synthases Alkyldihydroxyacetonephosphate synthase (AGPS) and Fatty acyl-CoA reductase 1 (FAR1) in peroxisome was inhibited, the cells would exhibit the resistance to ferroptosis until they were re-expressed. In another study, Cui et al. established the regulatory pathway of ferroptosis involving FAR1 as FAR1-ether lipid-TMEM189 (77). FAR1 promotes ferroptosis by reducing fatty acids to generate fatty alcohols necessary for synthesizing alkyl ether lipids and acetal phospholipids (77). Cui found that acetal phospholipids generated by plasmanylethanolamine desaturase (transmembrane protein 189, TMEM189) inhibited the expression of FAR1 and subsequent ferroptosis (78). Cui had characterized the inhibitory effect of TMEM189 on ferroptosis in different cancer cells. In contrast, Zou claimed that TMEM189 was not involved in ferroptosis regulation. The two contrary results may be explained by different cancer cell lines used in the experiment that expressed discrepant levels of TMEM189. This indicates that the level of TMEM189 protein in different cancer cells results in differences in the regulatory network between cancer cells. Further studies are needed to determine whether the differences in TMEM189 protein levels among varying cancer cell lines are regulated at the gene level, transcription level, or post-translational modification level, which may also contribute to elucidating the role of TMEM189 in ferroptosis.

### UPS

The ubiquitin-proteasome system (UPS) is closely linked to ferroptosis through targeting protein for degradation. The tumor suppressor BRCA1-associated protein 1 (BAP1), a member of the UCH family of deubiquitinase, is negatively associated with many tumors (79–81). BAP1 acts on system Xc^-^ and cleaves the mono-ubiquitin from lysine 119 of histone 2A (H2AK119Ub) in the SLC7A11 gene, thereby inhibiting the transcription of SLC7A11 and resulting in reduced cystine transport, decreased GSH production, lipid peroxide accumulation, and ultimately promoting the occurrence of ferroptosis (82, 83). Further research has demonstrated that the polycomb repressive complex 1 (PRC1), as the main E3 ubiquitin ligase of H2AUb, can enhance the binding of H2AUb to the SLC7A11 promoter and synergistically inhibit the expression of SLC7A11 with BAP1 (84). In addition, mono-ubiquitination of lysine 120 of histone 2B (H2BK120Ub) activates SLC7A11 expression, while p53 reduces H2BK120Ub level *via* promoting nuclear translocation of the deubiquitinase USP7, and finally promoting the occurrence of ferroptosis (85). Studies have shown that SLC7A11 is also positively regulated by deubiquitinase, and OTUB1, a deubiquitinase, can interact directly with SLC7A11 and repress ferroptosis by stabilizing SLC7A11. When the OTUB1 was inactivated, SLC7A11 levels diminished, and ferroptosis was promoted. It was also found that the cluster of differentiation-44 (CD44) could enhance the interaction of OTUB1 with SLC7A11 and prevent ferroptosis (86).

**Figure 4:**
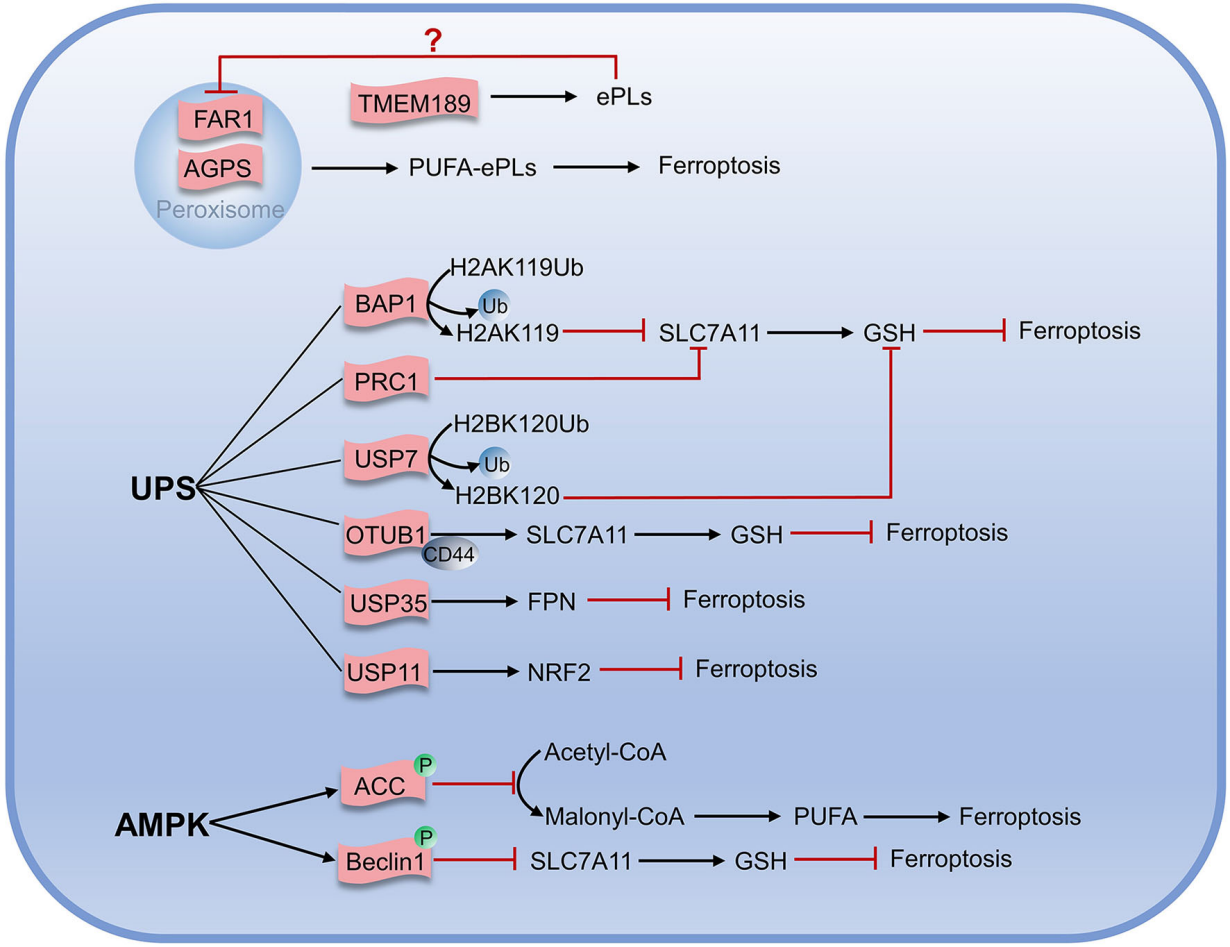
Metabolic regulation associated with ferroptosis.

It has been demonstrated that deubiquitinase USP35 is overexpressed in human lung cancer tissues and cell lines. Meanwhile, knockdown of USP35 can promote the degradation of FPN in lung cancer cells and reduce iron exportability that makes cancer cells sensible to ferroptosis, enhancing the chemotherapy effect on lung cancer cells (87). USP11 can stabilize NRF2 by deubiquitination, and USP11 inactivation can promote NRF2 degradation, making cells prone to ferroptosis and reproduction repressed (**Figure 4**) (88).

### AMPK

AMPK is an AMP-dependent protein kinase that regulates the generation and consumption of ATP to maintain intracellular energy homeostasis. Under energy stress, AMPK is activated and inhibits the physiological processes that consume energy directly or indirectly. Gan et al. recently identified a new mechanism on ferroptosis inhibition in which under energy-deficient conditions, activation of AMPK mediated phosphorylation of acetyl-CoA carboxylase (ACC) and ACC inactivation inhibited the conversion of acetyl-CoA to malonyl-CoA that can generate unsaturated fatty acids and participate in lipid peroxidation. Inhibiting the synthesis of unsaturated fatty acids would ultimately prevent ferroptosis (89–91). Beclin1, the first autophagy-associated protein discovered in mammals, acts as the core of the class III phosphatidylinositol 3-kinase (PtdIns3K) complex to promote the nucleation of autophagosomes (92). Song et al. found that Beclin1 induced ferroptosis independently of the PtdIns3K complex and could form Beclin1-SLC7A11 complex when phosphorylated at Ser90/93/96 by AMPK. This process directly inhibited the cystine transport function of system Xc^-^, leading to the accumulation of lipid peroxidation and ferroptosis increase (91, 93, 94). Overexpression of Beclin1 in cancer cells promoted the effect of system Xc^-^ inhibitors on ferroptosis and also promoted the effect of ferroptosis on anticancer *in vivo* (94). To sum up, AMPK has positive and negative regulatory roles in ferroptosis, so better understanding and accurately judging of the roles of AMPK may be of great but the far-reaching significance for target ferroptosis to treat tumors.

## Role of Ferroptosis in Hematologic Malignancies Treatment

### Leukemia

Leukemia is a malignant disease arising from the unlimited proliferation of hematopoietic stem cells, as malignant clones of cells could hinder the normal function of hematopoietic cells and affect the development of non-hematopoietic cells. Compared with other tumor cells, leukemia cells display higher transferrin expression and iron content, which causes the accumulation of ROS in leukemia cells more easily and ferroptosis occurs after cells undergo irreparable peroxidative damage. Thereby, promoting ferroptosis *via* further enhancing the iron content in leukemia cells seems to become a feasible strategy for leukemia treatment, which is also one of the hotspots in this field (95–97).

It has been found that Dihydroartemisinin (DHA), a derivative of the natural drug artemisinin, can induce ferroptosis in acute myeloid leukemia (AML) cells, owing to the capability of activating the phosphorylation of AMPK. Subsequently, AMPK inhibits the mTOR pathway and promotes autophagy, leading to autophagy-dependent degradation of FTH protein and the release of large amounts of free iron, which ultimately induces ferroptosis in AML cells (98). RSL3, as a small molecule inhibitor targeting GPX4, can also trigger a variety of programmed cell deaths, including ferroptosis in AML cells, and enhances the tumor suppressor effect of first-line chemotherapy drugs (cytarabine and adriamycin) on AML cells (99). Similarly, Balasubramanian et al. found that NRF2 may be a new target for the treatment of AML. Using Brusatol, an inhibitor of NRF2, could reduce the ability of NRF2 to eliminate ROS and increase the sensitivity of cytarabine and daunorubicin to AML (100). Ferroptosis induced by iron loading in leukemia cells exert a therapeutic effect, while the occurrence and poor prognosis of leukemia have been identified to be related to intracellular iron accumulation, such as inhibition of red blood cell differentiation when the iron is excessive (101). Therefore, much effort was focused on reducing of the amount of iron in cells. For example, iron chelating agents (DFO and DFX) have been used clinically to reduce the intracellular iron load (102). There are currently relatively few studies on treating leukemia with ferroptosis, and more experiments are required to elucidate the relationship between ferroptosis and leukemia treatment.

### Lymphoma

Diffuse large B-cell lymphoma (DLBCL) is one of the most frequent lymphatic malignancies, with the highest incidence in the non-Hodgkin lymphoma (NHL) family (103, 104). Since Cystine is a crucial negative regulator in the ferroptosis system, the lack of cystine in cells lead to the inhibition of GSH production, which ultimately gives rise to redox imbalance and ferroptosis. Therefore, the inability of lymphocytes to synthesize cystine has been regarded as a breakthrough point for the treatment of lymphoma. At an early stage, Gout et al. revealed that sulfasalazine could be used as a system Xc^-^ inhibitor to significantly reduce the growth of DLBCL in the abdominal cavity of rats (105). Stockwell et al. optimized imidazole-ketone-erastin (IKE) with stronger metabolic stability and water solubility, based on canonical system Xc^-^ inhibitor erastin. IKE could inhibit the growth of DLBCL in mice with more potent therapeutic effects through nanoparticle delivery (106). What’s more, Stockwell et al. indicated that inhibition of GPX4 activity also promoted the death of DLBCL cell lines (36). Consistently, GPX4 can reduce lipid peroxides and inhibit ferroptosis, and its overexpression has been demonstrated to be associated with poor prognosis in DLBCL patients (107).

### Multiple Myeloma

Multiple myeloma (MM) is a malignant proliferative disease in plasma cells, accounting for 10% of hematological malignancies (108). MM is associated with the accumulation of atypical plasma cells in bone marrow, abnormal production of monoclonal immunoglobulin, and increased serum calcium levels (109). GPX4 and SLC7A11 are key regulators of ferroptosis and are highly expressed in MM cells. A novel immunosuppressant Fingolimod (FTY720), can promote ferroptosis by reducing GPX4 and SLC7A11 mRNA and protein levels in U66 cells, enhancing ferroptosis and autophagy through the PP2A/AMPK pathway (110). In MM cells, high proteasome activity is the determinant for degrading misfolded immunoglobulin to ensure expected survival. As a proteasome inhibitor, Bortezomib has been clinically used to treat MM patients (111). However, the autophagy process activated by the accumulation of misfolded immunoglobulin in cells exhibits the resistance of MM against Bortezomib (112). Studies have shown that iron exposure can reduce the activity of the proteasome, hence increasing the efficacy of Bortezomib and Carfilzomib (the second generation of proteasome inhibitors used for MM therapy) in MM cells and leading to severe MM cell death by promoting ferroptosis (113). In order to overcome the drug resistance of Bortezomib, docosahexaenoic acid or eicosapentaenoic acid in combination with Bortezomib were used to enhance the sensitivity of MM cells to Bortezomib (**Table 1**) (114). Specifically, docosahexaenoic acid and eicosapentaenoic acid can modulate the redox balance in MM cells by reducing the content of GSH in MM cells, thus improving the therapeutic effect of Bortezomib. These combined therapeutic results provide a novel theoretical basis and therapeutic schedule for overcoming MM resistance to Bortezomib and further benefit clinical treatment.

**Table 1:** Inducers or inhibitors of ferroptosis in hematologic malignancies.

Cancer	Inhibitors/Inducers	Targeted sites
Leukemia	DHA	AMPK
	RSL3	GPX4
	Brusatol	NRF2
	DFO; DFX	Fe2+
Lymphoma	Sulfasalazine	system Xc-
	IKE	system Xc-
	RSL3	GPX4
Multiple myeloma	FTY720	GPX4; SLC7A11
	Bortezomib; Carfilzomib	Proteasome
	Docosahexaenoic acid; Eicosapentaenoic acid	GSH

## Conclusion and Perspective

In recent years, with the deep research on ferroptosis, several pathways other than GPX4-GSH have been identified to modulate lipid peroxidation directly. Meanwhile, large amounts of experiments have also been conducted to elucidate the roles of ferroptosis in hematological malignancies. However, many questions in the ferroptosis field remain to be addressed. For example, to what extent does lipid peroxidation lead to cell rupture and ferroptosis? Are other lipid peroxidation regulatory pathways associated with GPX4-GSH synergistic or merely complementary to GPX4? How can ferroptosis be used as a target of cancer therapy further (In **Table 2** we summarize the inhibitors or inducers associated with ferroptosis)? How to avoid the side effects of ferroptosis-related drugs (e.g., the toxic side effects of increased iron intake in leukemia treatment)? In conclusion, as a new modality of regulatory cell death, ferroptosis brings new possibilities for cancer treatment. However, further research is required for elaborating the mechanism of ferroptosis and its implications in cancer, which is of great scientific significance.

**Table 2:** Molecular compounds that regulate ferroptosis.

	Molecular compound	Targeted sites	Function
Inhibitor	α-Tocopherol	PUFA-ROO·	Blocks the lipid peroxidation caused by Fenton reaction
	Vitamin E	PUFA-ROO·	Blocks the lipid peroxidation caused by Fenton reaction
	Liproxstatin-1	PUFA-ROO·	Blocks the lipid peroxidation caused by Fenton reaction
	Ferrostatin-1	PUFA-ROO·	Blocks the lipid peroxidation caused by Fenton reaction
	DFO	Fe^2+^	Consumption of iron
	DFX	Fe^2+^	Consumption of iron
	CoQ_10_	Lipid peroxidation	Repairs lipid peroxide
Inducer	Erastin	system Xc^-^	Prevents cystine from entering cells
	IKE	system Xc^-^	Prevents cystine from entering cells
	Sulfasalazine	system Xc^-^	Prevents cystine from entering cells
	Sorafenib	system Xc^-^	Prevents cystine from entering cells
	FIN56	GPX4	Induces GPX4 degradation
	RSL3	GPX4	Covalently inhibits GPX4, leading to accumulation of lipid peroxides
	iFSP1	FSP1	Consumption of CoQ10 leads to a decrease in GPX4 activity
	MTX	DHFR	Inhibits DHFR activity and reduce BH4 production
	Docosahexaenoic acid	GSH	Consumption of GSH
	Eicosapentaenoic acid	GSH	Consumption of GSH
	Brequinar	DHODH	Decreases DHODH activity and resultes in accumulation of mitochondrial peroxide lipids

## Author Contributions

FW and HL conceptualized the study. FW oversaw the literature review involved in all aspects of designing and writing the manuscript. FW and HL performed the literature review. FW, ZM, and HL wrote the manuscript and designed the figures. ZZ, YG, and ZM provided input on the discussion of various sections. All authors contributed to the article and approved the submitted version.

## Funding

This work was supported by the National Natural Science Foundation of China Grants 31770827 and 21736002 to FW, 31870791 and 91753205 to ZM.

## Conflict of Interest

The authors declare that the research was conducted in the absence of any commercial or financial relationships that could be construed as a potential conflict of interest.

## Publisher’s Note

All claims expressed in this article are solely those of the authors and do not necessarily represent those of their affiliated organizations, or those of the publisher, the editors and the reviewers. Any product that may be evaluated in this article, or claim that may be made by its manufacturer, is not guaranteed or endorsed by the publisher.
